# Energetic mitochondrial failing in vitiligo and possible rescue by cardiolipin

**DOI:** 10.1038/s41598-017-13961-5

**Published:** 2017-10-20

**Authors:** Maria Lucia Dell’Anna, Monica Ottaviani, Daniela Kovacs, Simone Mirabilii, David A. Brown, Carlo Cota, Emilia Migliano, Emanuela Bastonini, Barbara Bellei, Giorgia Cardinali, Maria Rosaria Ricciardi, Agostino Tafuri, Mauro Picardo

**Affiliations:** 1 Cutaneous Physiopathology Lab, San Gallicano Dermatologic Institute, IFO IRCCS Rome, via Elio Chianesi, 53 00144 Italy; 2 Department of Clinic and Molecular Medicine Faculty of Medicine and Psychology, La Sapienza University, via Rovigo 1 Rome, 00162 Italy; 3 Department of Human Nutrition, Foods, and Exercise, Virginia Tech, 1981 Kraft Drive, Virginia Tech Corporate Research Center, Blacksburg, VA 24060 USA; 4 Istopathology, San Gallicano Dermatologic Institute, IFO IRCCS Rome, via Elio Chianesi, 53 00144 Italy; 5 Plastic Surgery, San Gallicano Dermatologic Institute, IFO IRCCS Rome, via Elio Chianesi, 53 00144 Italy

## Abstract

Vitiligo is characterized by death or functional defects of epidermal melanocytes through still controversial pathogenic process. Previously, we showed that mitochondria-driven pre-senescent phenotype diminishes the capability of vitiligo melanocytes to cope with stressful stimuli. In the current study, we investigated markers of mitochondrial energy metabolism including the PGC1a axis, and then we determined the index of mitochondrial impairment using a cytomic approach. We found in cultured epidermal vitiligo melanocytes, compared to healthy ones, low ATP, increased proton leakage, and altered expression of several glycolytic enzymes (hexokinase II, pyruvic dehydrogenase kinase 1 and pyruvic kinase M2), We suggest that the low ATP production may be sufficient in steady-state conditions but it is unable to cover further needs. We also found in vitiligo melanocyrtes hyper-activation of the PGC1α axis, finalized to counteract the energy defect. Cytomic analysis, supported by MitoTracker Red pattern and *ex-vivo* immunohistochemistry, suggested an increased mitochondrial mass, possibly useful to ensure the essential ATP level. Finally, pharmacological cardiolipin stabilization reverted the energetic impairment, confirming the initial mitochondrial role. In conclusion, we report new insight in the pathogenetic mechanism of viitligo and indicate that the mitochondrial failure rescue by cardiolipin manipulation may be a new intriguing target in treatment development.

## Introduction

Vitiligo is a depigmenting disease characterized by white skin lesions due to the functional loss of melanocytes. The pathogenic process leading to the melanocyte defect is still debated. Concurrent events, including autoimmune and metabolic processes, may occur^1–6^. Until recently, most of the focus has been on the final events causing the clinical manifestations, i.e., the immune-mediated damage, without considering the initial detrimental process, possibly related to an intrinsic melanocyte defect. Moreover, current *in vitro* studies on vitiligo pathogenesis have employed predominantly normal or immortalized or neonatal melanocytes, even murine cells, and examined the potential alterations by inducing an injury in these cells. This is presumably similar to that present in vitiligo melanocytes. However, few studies have performed functional and morphological evaluations directly of cultured vitiligo cells under basal conditions.

Previously, intrinsic functional melanocytes and keratinocytes defects have been demonstrated in vitiligo. In particular, the loss of redox balance (high spontaneous ROS generation and deficit of the antioxidant network) can be considered a hallmark of vitiligo, where defective mitochondria appear to be prime candidates for such alterations^6–10^.

We previously demonstrated some structural and functional alterations affecting vitiligo cells, independently of the ontogenetic features, and defined the mitochondrial involvement in disease pathogenesis. Accordingly, high ROS production was affected by Cyclosporin A(CsA), which targets mitochondrial transition pores, the transmembrane potential (ΔΨ_m_) was lost, the expression of some Electron Transport Chain (ETC) proteins was altered and susceptible to mild stress, the activity of the ETC Complex I (CxI) was defective, the transmembrane distribution of cardiolipin, which accounts for accurate ETC arrangement, was deregulated, and the cells were extremely susceptible to the specific mitochondrial inhibitor Rotenone^7–10^. Thereafter, our studies have provided evidence for the occurrence of a degenerative process involving melanocytes from an apparently healthy area, which is characterized by high p53 expression and senescent secretome^11^. Our hypothesis is that vitiligo melanocytes may be affected by a degenerative process, as before documented, associated with mitochondrial impairment according to the relationship between mitochondrial status and degenerative events.

In the present study, we asked whether in vitiligo melanocytes mitochondria are able to carry out the main specific activity, the ATP production, whether they activate compensatory mechanisms, and whether alternative substrate or membrane stabilization may rescue the defective activity. In particular, we asked whether mitochondria are involved in the vitiligo cellular impairment because they represent the main site of a generic ROS production^12,13^ or whether they are implicated due to an intrinsic defect affecting the multistep process resulting in ATP production starting from glucose intake to its metabolism and final delivery to ETC. Beside the evaluation of some key enzymatic steps of glucose utilization, in our *in vitro* model we examined the underlying cross-talk between mitochondria and the nucleus^14–18^. Finally, according to previously detected mitochondrial membrane defects, mainly the altered transmembrane distribution of cardiolipin and altered expression of some ETC complexes, we searched for further evidence supporting the role of membrane cardiolipin in such alterations.

Now, we describe how the previously reported altered arrangement of the ETC proteins affects the ATP level and subsequent mitochondria-nucleus cross-talk by activating some key mediators, including PGC1α (peroxisome proliferator gamma coactivator 1), resulting in increased compensatory mitochondrial mass. This study describes the potential modulation of the energetic pathways by small molecules that are able to interfere with mitochondrial lipid membrane assessment, suggesting that the functional and energetic defects occurring at the mitochondrial level may be the initial step leading to the defective functional profile.

## Results

### Failure of Energetic metabolism in primary cultured vitiligo melanocytes

**Figure 1:**
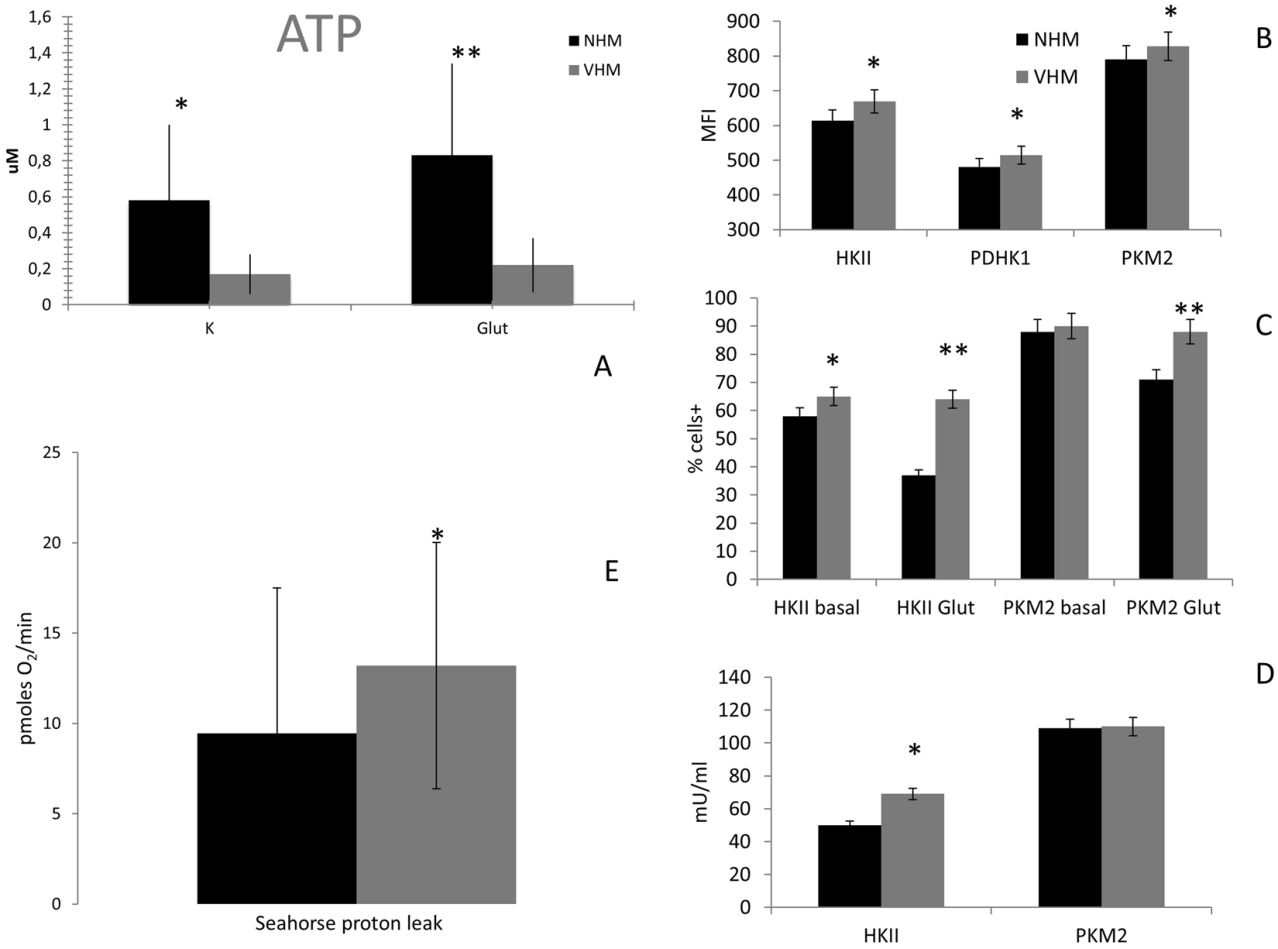
ATP production in NHM and VHM in basal and forced mitochondrial activity. (**A**) Quantification has been performed by fluorimetric kit. It was carried out in basal condition (standard culture medium) and after 12 days treatment with 20 mM L-glutamine. Data are mean ± SD of all the performed experiments. *p = 0.008; **p = 0.009. (**B**) Flow cytometric analysis of MFI for HKII, PKM2, and PDHK1. (**C**) The percentage of positive cells for HKII and PKM2 was also evaluated and an increased expression was observed in VHM in basal condition; NHM down-regulate its expression after L-glutamine supplementation whereas VHM aren’t affected by the presence of the alternative substrate, underlying the difference between the two populations. (**D**) The activity of HKII was also forced in a compensatory strategy. Data are mean ± SD of all the performed experiments. *p = 0.05; **p = 0.01. (**E**) A relevant proton leak was detected in VHM, underlying the inability of ETC to correctly finalize the specific activity. Consequently, the mitochondrial performance, represented by BHI, was negatively affected. BHI is calculated as (ATP × Reserve Capacity)**/**(proton leak × non mitochondrial respiration). Data are mean ± SD of all the performed experiments. *p = 0.01.

Mitochondria impairment is associated with some harmful cellular situations, including senescence, autophagy, and death^14–20^. To define the biological relevance of previously reported mitochondrial defects, we searched for some potentially related metabolic alterations. First, we tested the final, but not unique, product of the mitochondrial activity, namely, ATP^12,14^. VHM showed a significant reduction in ATP production (0.17 ± 0.11 μM vs 0.58 ± 0.42 μM; p = 0.008) compared to NHM maintained in standard culture conditions, according to the hypothesized initial metabolic defect of apparently healthy vitiligo skin (Fig. 1A).

Looking for the possible defective step in the ATP production, we searched for the expressions and activities of some key enzymes involved in energetic metabolism that are upstream of the ETC system (i.e. the glucose metabolism). The expressions of HKII (hexokinase II), PDHK1 (pyruvic dehydrogenase kinase 1), and PKM2 (pyruvic kinase isoform M2) were higher in vitiligo cells compared to normal ones. In particular, the Mean Fluorescence Intensity (MFI) of VHM versus NHM for HKII was 669 versus 614. For PKM2, the value was 828 versus 790, and for PDHK1, the value was 514 versus 481 (Fig. 1B). The percentage of HKII-positive cells was also increased in VHM (65% vs 58%) (Fig. 1C). These data suggest that VHM are characterized by an impaired energetic metabolism, with the defective ATP production compensated by an increased activity of enzymes involved in glucose utilization.

### The supply of an alternative substrate does not improve the mitochondrial performance

Next, we evaluated whether the impairment of mitochondrial functionality could be recovered by the addition of L-glutamine, an agent that is able to strengthen mitochondrial activity, providing an alternative substrate for the Krebs cycle^21–24^. The addition of L-glutamine reduced the percentage of positive cells for HKII (from 58% ± 5 to 37% ± 7 positive cells, p = 0.01) and PKM2 (from 88% ± 10 to 71% ± 6 positive cells, p = 0.01; its basal value was similar in VHM and NHM) in NHM but not in VHM, where the modification was not relevant, potentially indicating a defective ability to take advantage of alternative substrates. The *in vitro* exposure to L-glutamine intensified the ATP production in NHM (from 0.58 ± 0.42 to 0.83 ± 0.51 μM, p < 0.05), whereas in VHM the ATP levels are only slightly modified (from 0.17 ± 0.11 μM to 0.22 ± 0.16 μM; p = 0.009 between normal and vitiligo samples). We conclude from these results that the enzymes of Krebs cycle are not responsible for the ATP low level and then we suggest downstream steps.

### Further evidence for the proper activity of glucose pathway

We observed that the HKII activity, which accounts for glucose phosphorylation, and is thus essential for its subsequent utilization, was higher in VHM than in NHM (69 mU/ml vs 50 mU/ml), whereas PKM2 activity was not significantly different (Fig. 1D).

**Figure 2:**
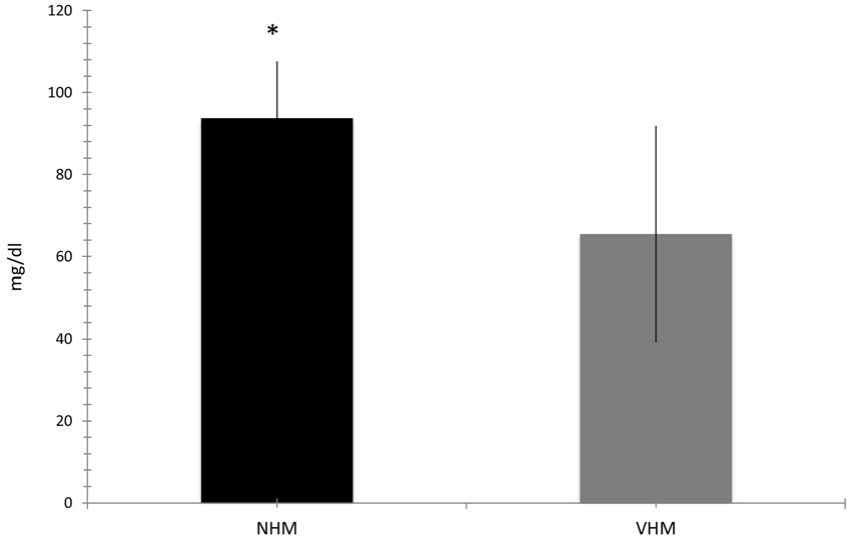
Glucose uptake. Spectrophotometric assay defined increased glucose uptake by the defined medium according to the increased expression and activity of HKII. Data are mean ± SD of all the performed experiments. *p = 0.05.

Simultaneously, the amount of glucose in the medium was tested and, at comparable culture passage and degree of confluence, the NHM showed lower, when compared to VHM, utilization of the glucose provided by the defined medium (117 mg/dl in M254). In particular, after 3 days of standard culture, we found 93.75 ± 13.85 mg/dl in the culture medium of normal melanocytes vs 65.6 ± 26.35 mg/dl of vitiligo cells (p = 0.002) (Fig. 2).

### Alteration of the Bionenergetic health index in vitiligo melanocytes

Consistent with this finding, we suggest that VHM, despite the increased uptake of glucose and expression of enzymes devoted to glucose utilization, are unable to properly maximize the final pathway resulting in ATP production. To confirm the ineffective mitochondrial function, we used the real time method. Primary melanocytes (VHM = 6 vs NHM = 6) were assayed for metabolic activity^19^ using a plate respirometry approach and the Seahorse XF analyzer. We evaluated ATP-linked respiration, spare respiration, basal respiration, proton leak, maximal respiration and the derived parameters represented by the Bioenergetic Health Index (BHI)^19^.

A statistically significant increase of proton leak was revealed in the VHM population (Fig. 1E) (13 vs 9, p < 0.01); it determines the reduction of BHI (from 7.7 of NHM to 6.4, p < 0.01) indicating a partial inability of vitiligo cells to properly finalize the ETC activity. This BHI loss was observed even if the isolated parameters, excluding proton leak, were not significantly different between the two populations (Table 1) and is characterized by high standard deviations. Using the real time evaluation, the detection of ATP did not fit with that quantified using the standard method, most likely due to the elevated standard deviation observed. Overall our data suggest the incorrect functionality of the respiration machinery fitting with high ROS production and low ATP production of VHM.

**Table 1:** Seahorse analysis data. Media and standard deviation of the different evaluated parameters by Seahorse approach.

	Basal Rate	Maximal Respiration	Proton Leak	ATP	Spare Respiration
VHM	102 ± 35	201 ± 155	13 ± 7	92 ± 32	99 ± 80
NHM	76 ± 50	149 ± 135	9 ± 8	73 ± 45	73 ± 88

### Adaptive Intracellular signaling around mitochondria

When the metabolic activity is compromised, specific pathways have to be activated in order to optimize it^18,25–31^. Therefore, we investigated intracellular signalling pathways underlying the metabolic adaptation of the cells to the mitochondrial defects, such as the mitogenesis-related PGC1α axis and CREB (cAMP responsive element binding protein) pathway^32–37^.

These analyses confirmed our previous data on the higher degree of CREB phosphorylation^11^ in VHM compared to NHM (MFI 260 ± 10 vs 200 ± 16, p < 0.05). Furthermore, FAK^y397^ (focal adhesion kinase phosphorylated in Tyrosine 397) (MFI 650 ± 32 vs 600 ± 18, p < 0.05) and FAK^s910^ (FAK phosphorylated in Serine 910) (MFI 428 ± 25 vs 355 ± 17, p < 0.05) displayed higher phosphorylation levels in VHM compared to NHM. In addition, the percentage of cells with JNK phosphorylated, which probably target mitochondria, was higher in VHM compared to NHM (30 ± 4% vs 19 ± 7%). No significant alteration in the STAT1 pattern was observed (data not shown).

**Figure 3:**
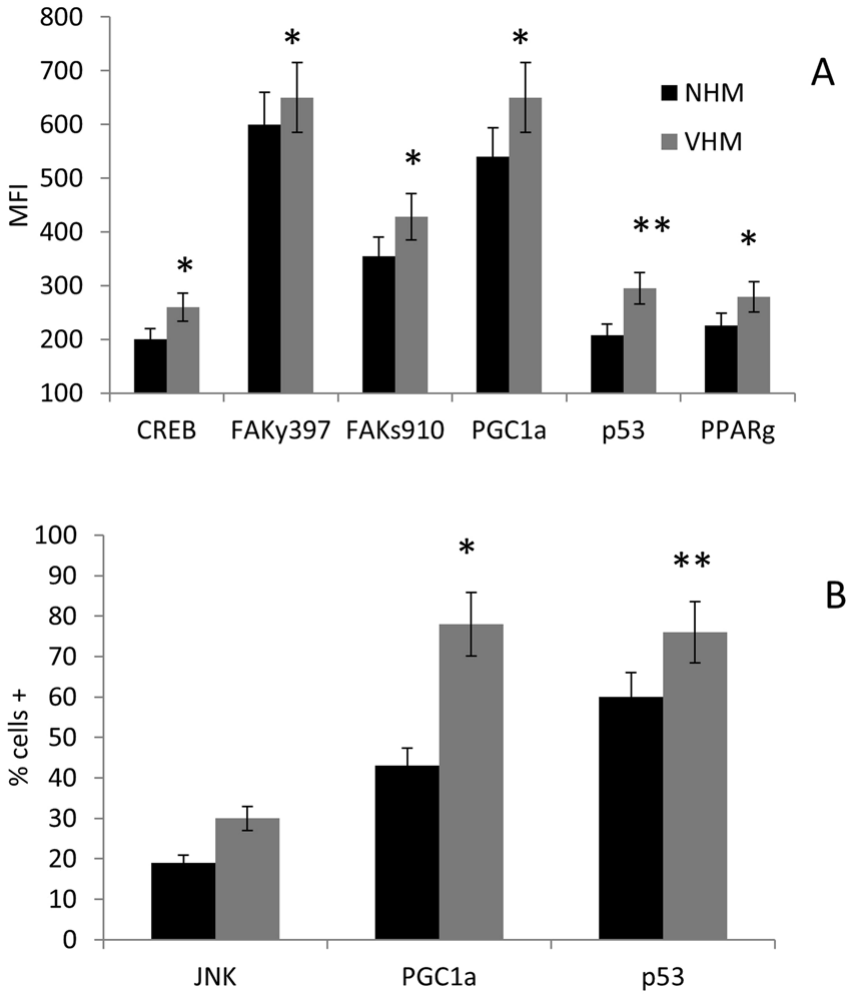
How the cross-talk mitochondrion-nucleus may be affected. Flow cytometric analysis of some key factors of the intracellular network confirmed the increased phosphorylation of CREB and revealed a parallel higher phosphorylation of FAK which, together with the increased expression of PGC1a p53 and PPARg, testified for the increased mitogenesis in an attempt to improve mitocohondrial activitiy. Both MFI and percentage of positive cells were increased in VHM population. Results are referred to analyses carried out in basal condition. Data are mean ± SD of all the performed experiments. *p = 0.05; **p = 0.01.

Interestingly, the expression of PGC1α was also higher in VHM compared to NHM (MFI 650 ± 23 vs 540 ± 18; p < 0.05), inversely correlated with ATP content and positively correlated with glucose utilization (Fig. 3A,B). The evaluation of p53 expression confirmed in VHM the increased protein levels ads previously demonstrated^11^ (MFI 295 ± 21 vs 208 ± 27; p < 0.001). The induction of PGC1α was sustained by the parallel over-expression of PPARγ (peroxisome proliferator activated receptor gamma). These results suggest that vitiligo melanocytes need the over-expression and activity of the PGC1a pathway to ensure the essential ATP production.

### Evidence for mitochondrial mass increase in viitligo melanocytes

The next question was whether the hyperactivation of the mitogenenesis also accounted for enlargement or increased quantity of mitochondria per cell. The occurrence of this morphological evidence may be considered further proof of the adaptive cellular mechanism. Accordingly, NHM and VHM were cultured in complete medium under standard conditions and were detached immediately before the flow cytometric acquisition to avoid any interference with the cellular physical features. No staining or treatment was performed.

**Figure 4:**
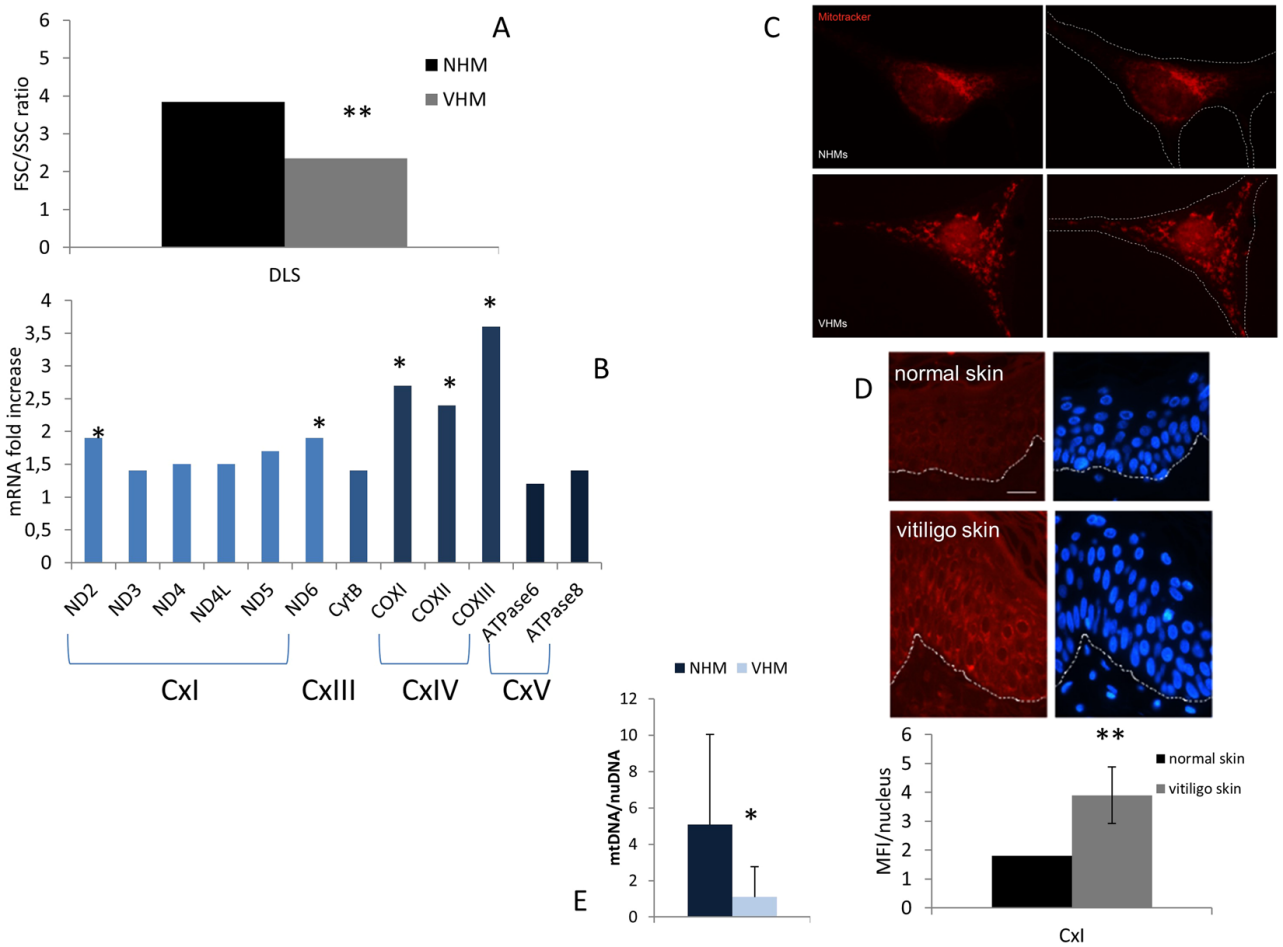
Multiparametric analysis of mitochondrial mass. (**A**) Cytomic approach, based on the DLS parameter, demonstrated increased mitochondrial mass. It was further explored by alternative methods: (**B**) mRNA for some mitochondrial proteins was increased; (**C**) analysis of Mitotracker signal by fluorescence microscope underlined an enlarged structure and a specific organization of mitochondria in VHM; (**D**) signal for CxI expression obtained by immunohistochemistry staining was increased. Finally, (**E**) mtDNA quantitation, performed using nuclear DNA as internal reference, confirmed the increased volume of mitochondria. Data are mean ± SD of all the performed experiments. *p = 0.02; **p = 0.05.

Differential Light Scatter (DLS) analysis^38–42^ revealed that the forward/side scatter ratio was significantly lower in VHM with respect to the NHM (2.35 vs 3.84, p < 0.05), suggesting increased mitochondrial mass, potentially due to an enlargement of the mitochondria (Fig. 4A). Accordingly, we found increased expression in VHM of some mitochondrial genes (ND2, ND5, ND6 of CxI, and COXI, COXII, COXIII of CxIV) (Fig. 4B), without an increase in mitochondrial DNA content (mtDNA/nuDNA 1.09 VHM vs 5.08 NHM, p = 0.03) (Fig. 4C), confirming the increased mitochondrial volume rather than the number. MitoTracker staining revealed that the mitochondria were widely distributed throughout the cytoplasm as spherical and enlarged structures and less regularly interconnected in VHM compared to NHM. Even if the statistical analysis lacked a significant value, the quantitative analysis of the mitochondrial area indicated an increase in VHM (+24%) (Fig. 4D). Moreover, *ex vivo* immunohistochemical quantitative analysis of CxI 15 kDa expression on skin sections revealed a significant increase in vitiligo epidermis compared to healthy epidermis (MFI 3.9 ± 2 vs 1.8; p < 0.05), which confirms our previous data [8] (Fig. 4E).

Our results suggest that the impaired functionality of mitochondria in vitiligo results in a compensatory increase in mitochondrial mass to ensure at least the minimal energy production in steady-state conditions. The observation carried out in *ex vivo* samples strongly enforced the translational relevance and clinical value of the *in vitro* study.

### Stabilization of membrane arrangement improve mitochondrial functionality in vitiligo melanocytes

Our initial data suggested that the transmembrane distribution of cardiolipin is altered^9,10^ in vitiligo cells. Our current data indicated that, independent of substrate availability and specific glycolytic enzymatic activities, vitiligo mitochondria are not able to produce normal amounts of ATP, resulting in a compensatory increase in mass. Thus, we asked whether the incorrect transmembrane distribution of cardiolipin might account for the reported energy loss and whether a small molecule^43–45^ could stabilize the transmembrane cardiolipin pattern, which may counteract this defect and reverse the dys-functional phenotype. MTP-131 serves as a cap for cardiolipin and stabilizes it. Consequently, the transmembrane arrangement of ETC multiprotein complexes improves the mitochondrial activity, starting from the paradigmatic one^43–46^.

**Figure 5:**
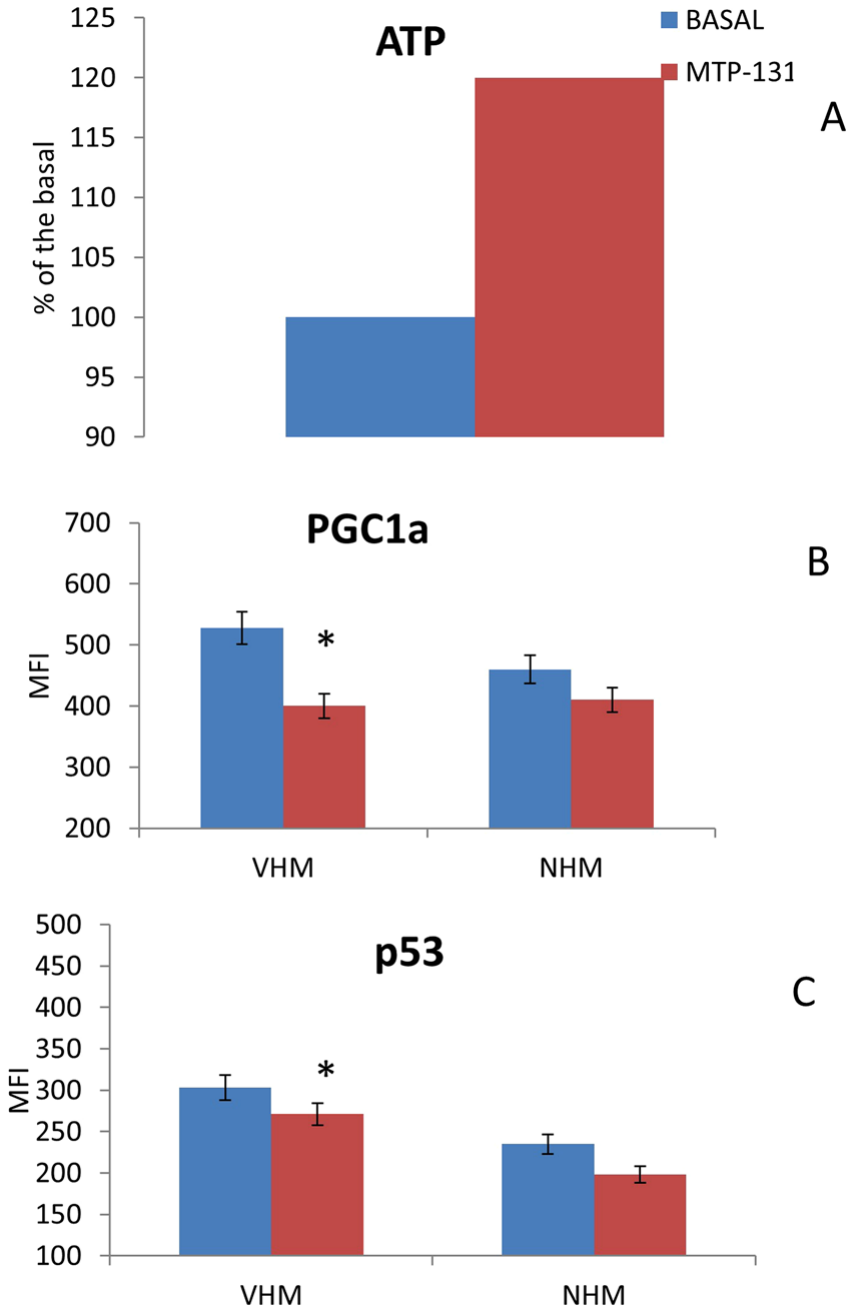
Cardiolipin stabilization may improve mitochondrial performance. The *in vitro* treatment of cells with 2μM MTP-131 for 7 days rescued the ability of VHM to (**A**) produce ATP. Consequently, the expression of (**B**) PGC1a and (**C**) p53 was reduced. Data are mean ± SD of all the performed experiments. *p = 0.05.

We observed that *in vitro* treatment of VHM with MTP-131 restores ATP levels (120% of the basal) and thus reduces PGC1α (MFI from 521 to 400) and p53 (MFI from 303 to 271) expression (Fig. 5). This experimental result suggest us that the mitochondrial impairment is an early event in the overall cellular defect occurring in vitiligo opening new perspectives in therapeutical approaches.

## Discussion

Our *in vitro* study demonstrates that during vitiligo (a) VHM are characterized by an impaired energetic metabolism, where the defective ATP production is tempted to be compensated by an increased activity of enzymes involved in glucose utilization, (b) supplying the cells with alternative substrate did not improve the energetic level, and (c) the stabilization of lipid components of the mitochondrial membrane rescues the affected activities and pathways.

Viitligo represents a complex disease where multiple pathways lead to cellular detriment. Previous data motivated further inquiry on the role of mitochondria in this impairment^1–10,47–51^.

Here, we demonstrated that vitiligo mitochondrial impairment manifests as reduced ATP production whereas the analyzed enzymes related to the Krebs cycle appear to be more represented, suggesting a compensatory activity of the upstream metabolism that provides the substrates for ETC. Consistent with these findings, VHM show an enhanced cellular uptake of glucose from the culture medium, but the final ATP levels are still the lowest. In addition, VHM is characterized by high proton leak, supporting the idea that the ETC does not appropriately function and a shift in focus is needed from supply availability to final utilization. Proton leak belongs to the parameters (ATP-linked respiration, proton leak, maximal respiration, reserve capacity and non-mitochondrial respiration) that are defined by the BHI. Highest proton leak may represent an attempt from vitiligo cells to protect themselves from high ROS production^52^, or alternatively, this increased leakage across mitochondrial membrane may be directly caused by ROS^53^. Nevertheless, this coupling inefficiency results in decreased ATP production, affecting the bioenergetics status of vitiligo cells and is supported by the reduction of the BHI. Accordingly, VHM are poorly responsive to an external supply of L-glutamine, which can elevate ATP levels in NHM where the ETC properly functions.

To determine the functional value of the mitochondrial impairment featuring vitiligo cells, we dissected some steps of the intracellular signaling pathway involving mitochondria. The focal adhesion kinase, FAK, regulates different metabolic processes, such as adhesion, proliferation, and differentiation via the selective phosphorylation of different sites. Its auto-phosphorylation at Tyr^397^ appears to be critical to maintain cellular proliferation via the recruitment of Src kinases and subsequent further FAK phosphorylation and activity enhancement^54,55^. FAK phosphorylation at Tyr^397^ exhibits a dual contrasting effect: it induces the phosphorylation and activation of JNK, and it promotes the nuclear translocation of FAK itself followed by its assembly with PGC1α. JNK, when activated, localizes to mitochondria via a membrane-embedded receptor and results in ΔΨ_m_ loss, ROS hyper-production, and lipoperoxidation^56^. The nuclear localization of PGC1α/FAK complex activates PGC1α, promoting mitochondrial biogenesis, resulting in a increased mitocohondrial mass, fitting reduced FSC/SSC ratio, increased mitochondrial area of MitoTracker distribution, increased mitochondrial mRNA for some ETC proteins, and CxI over-expression.

A key factor in mitochondrial control is PGC1α, and the complexity of PGC1α sensor for energy metabolism discrepancies, provides a clear visualization of the multiple signaling cross-talks in mitochondria. As previously reported in other *in vitro* systems^32^, dangerous stimuli activate FAK phosphorylation and promote its association with PGC1α.

Mitochondrial biogenesis, which is finalized to supply adequate ATP production, may also be sustained by CREB activation due to mitochondrial impairment.

When respiratory chain defects occur, the mitochondria produce higher ROS levels, thereby activating PGC1α and promoting, via PPARγ, mitochondria compensatory biogenesis^57^. In addition, p53 can directly bind to PGC1α, where the latter acts as a transcriptional activator. Moreover, p53, a cross-talk signaling molecule between mitochondria and the senescence process, controls adaptation to metabolic stress and increases OXPHOS function^33,58^.

These results prompted the hypothesis that increased mitochondrial mass could be tentative to compensate for defective activity, i.e., energetic impairment, via different convergent pathways.

Starting from previously and currently reported mitochondrial alterations, we examined the role of the membrane component cardiolipin. Drug-induced cardiolipin stabilization by MTP-131 recovered the energetic profile, mitochondrial structural and functional parameters, confirming that the impairment and metabolic defects observed in vitiligo melanocytes were due to the initial ETC defects.

In conclusion, according to our current data, we describe a mitochondrial energetic failing and the possible rescue by cardiolipin manipulation. A potential metabolic network can be depicted as follows: inappropriate mitochondrial structure/function, which is characterized by increased ROS production and inadequate ATP levels, may activate cross-talk between the nucleus and mitochondria via PGC1α and FAK, which aims to compensate via an increase in mitochondrial mass and potentially revert this metabolic impairment^59^. Regulation of this early dysfunction at cardiolipin level by small molecules stabilizing it, rescues the depicted mitochondrial impairment and it is predicted to play a role for the therapeutic control of vitiligo^6,60^. Further evidence supporting the part of the mitochondrial membrane stability in the functional performance of the cell power-house is provided by the role of another membrane interfering, the melatonin. Melatonin and its metabolites have been reported to improve the mitochondrial antioxidant and energetic functions by acting as electron donor in ETC and as cardiolipin stabilizing agent. Both the mechanisms stop the ROS excessive generation and recover ATP production^61^.

A corollary consideration of our data is that flow cytometric analysis of DLS may represent an easy and convenient method to identify functional borderline mitochondrial defects in primary cells. According to the light scattering studies of Shapiro^39^ on the relationship between DLS and cellular organization, we indeed described how mitochondrial mass can affect the FSC/SSC ratio in primary epidermal melanocytes.

## Materials and Methods

### Skin biopsies and Cell cultures

19 vitiligo and 25 normal subjects were included in the study. Vitiligo subjects were classified according to the VETF criteria^60^, and only those with the non-segmental vitiligo subtype were included. The control samples (normal human primary epidermal melanocytes, NHM) were obtained from subjects who underwent plastic surgery for diseases unrelated to pigmentation disorders. Primary epidermal melanocytes from vitiligo subjects (VHM) were isolated from 1 cm^2^ skin biopsy in non-lesional skin. Isolated NHM and VHM^9^ were cultured in 254 Medium (Cascade Biologics, ThermoFisher) supplemented with specific Growth Factors cocktail (Cascade Biologics) and penicillin/streptomycin (Gibco). All of the analyses were performed between 3 and 9 culture passages.

Skin biopsies from vitiligo (n = 5) and normal (n = 3) subjects were also paraffin-embedded for immunofluorescence analysis.

The institutional Ethical Committee (IFO-Fondazione Bietti) approved the study and all the experimental protocols. Informed consent was obtained from each subject before the start of the study. All the experiments were performed in accordance with the relevant guidelines and regulation.

### Treatments

VHM and NHM were treated for 12 days with 20 mM L-glutamine (Gibco) in complete medium. Alternatively, the cells were exposed for 7 days to 2 μM MTP-131 (StealthPeptides, Inc.), and the mitochondria-associated index and signaling were assayed.

### ATP determination

The intracellular level of ATP was measured using a commercial fluorimetric kit (ThermoFisher) according to the manufacturer’s instructions. The results were reported as μM mean value ±SD.

### Real time analysis of mitochondrial metabolic activity

Mitochondrial respiration rates were measured using the XF24 Extracellular Flux Analyzer (Seahorse Bioscience, Agilent M&M Biotech). Briefly, VHM and NHM cells were seeded onto XF24 plates (40000 cells/well) for 48 hours prior to the experiments. On the day of the analysis, Complete Defined Medium was replaced with unbuffered DMEM medium (Gibco) supplemented with 2 mM L-glutamine, 11 mM Glucose (Sigma Aldrich) and 1.2 mM Pyruvate (Sigma-Aldrich) adjusted to pH 7.35, and the plates were incubated for 30 min at 37 °C in a CO_2_-free incubator. Oxygen Consumption Rates (OCR) were measured for the basal state and following the sequential injection of oligomycin (1 μM), FCCP (0.6 μM), and a mix of Antimycin A (1 μM) and Rotenone (1 μM) (all from Seahorse MitoStress Test Bioscience kit) in each well. After the assay, cells were detached and manually quantified to assess the cell number and viability. The BHI was calculated according to the following formula: BHI = (ATP linked respiration × reserve capacity)/(proton leak × non-mitochondrial respiration).

### Glucose determination

The intracellular level of glucose was measured using a commercial colorimetric kit (Sigma Aldrich) according to the manufacturer’s instructions. The results were reported as mg/dl mean value ±SD.

### Glycolytic enzymes activity assay

The activities of the PKM2 (AbCam) and HKII (AbCam) glycolytic enzymes were measured using a commercial colorimetric kit according to the manufacturer’s instructions. The results were reported as U/mg protein ±SD. The test was performed with and without the *in vitro* addition of 20 mM glutamine.

### *Ex-vivo* immunofluorescence analysis

Serial sections derived from formalin-fixed and paraffin-embedded blocks were de-waxed in xylene and rehydrated through a graded series of ethanol. Tissue sections were incubated with mouse MoAb anti-Complex I 15 kDa (1:50; Molecular Probes, Life Technologies) and then visualized using a goat anti mouse-Texas Red 1:100 (Santa Cruz Biotechnology Inc.). Nuclei were counterstained with 4′,6′-diamidino-2-phenylindole (DAPI) (Sigma Aldrich). Fluorescence signals were analyzed using stained images with a CCD camera (Zeiss). The analysis was performed at 63x and 100x, and the results are shown here at 100x. Quantitative analysis of CxI fluorescence intensity was performed using AxioVision 4.7.1 software (Zeiss). The results were expressed as the fold increase of the fluorescence intensity reported as the mean value ± SD relative to the healthy skin, which was set as 1 by definition.

### Flow cytometry

All of the flow cytometric analyses were performed using FACSCalibur (Becton Dickinson) equipped with a 15 mW, 488 nm, air-cooled argon ion laser for excitation of FITC (FL1), PE (FL2), and PercP (FL3) and with a 10 mW, 635 nm, red diode laser for excitation of APC (FL4). The optical bench of the instrument was maintained in the standard configuration. The cytometry stability and sensitivity were confirmed before each acquisition session by measuring the intensity and the variation coefficient of scatters and fluorescence signals of the Nile Red microbeads (Becton Dickinson). The FL4 detection was optimized by time delay calibration using APC microbeads (Becton Dickinson). FL1-H, FL2-H, FL3-H height signals were collected after logarithmic amplification, while both FSC-H and SSC-H height signals were collected after linear amplification. The same saved setting was used for all of the samples, even when acquired in different sessions. For each sample 5,000 events were acquired. Samples were acquired and analyzed using CELLQuest 3.3 software (Becton Dickinson) for multiparametric data analysis.

For analysis of the intracellular signaling, the detached cells were fixed and permeabilized and then stained with specific MoAbs. Briefly, primary melanocytes were fixed with 2% paraformaldehyde diluted in PBS at 37 °C for 20 minutes, washed once with cold PBS (13 minutes, 4 °C, 1200 rpm), and then permeabilized with pre-frozen methanol 80% for 1 hr on ice or overnight at −20 °C. After permeabilization, the cells were washed once with PBS and stained with fluorochrome-conjugated mouse MoAbs anti-FAK^y397^(Invitrogen), anti-FAK^s910^, anti-CREB, anti-JNK, anti-STAT1 (all from Becton Dickinson), and with purified rabbit MoAbs anti-PGC1a (Abcam), anti-p53 (AbCam), and anti-PPARg (Santa Cruz Biotechnology) (30 minutes, 4 °C, dark) and once washed with PBS. For staining with purified MoAbs, a secondary goat anti-rabbit Alexa Fluor 647 (Cell Signaling) was used. The percentage of positive cells for specific Ab and its MFI (Median Fluorescence Intensity) were analyzed in the selected region, which was defined on the basis of DLS, excluding debris. The results were reported as the mean value ±SD.

For the analysis of the expression of the enzymes involved in energetic metabolism, the cells were fixed and permeabilized and then stained (30 minutes at 4 °C) with purified rabbit MoAb anti-PKM2, anti-HKII, ant-PDHK1 (all from Cell Signaling). After the wash, the cellular pellet was stained with Alexa Fluor 488 Goat anti-Rabbit (Cell Signaling) for an additional 30 minutes at 4 °C. The cellular pellet was resuspended in cold PBS, and the acquisition was immediately performed. The percentage of positive cells for each specific Ab and its MFI were analyzed in the DLS-defined region, excluding debris. The results were reported as mean value ±SD.

For the DLS study, the cells were gently detached, resuspended in culture standard medium and immediately analyzed without any further manipulation, including staining and/or fixation. All of the samples were acquired by setting the medium flow rate to avoid turbulence that could potentially affect the DLS. The forward/side scatter ratio was individually calculated for each cell belonging to the viable region, which was defined on the basis of the physical parameters, during the analysis and plotted as a distribution histogram on a linear scale^41^.

### Fluorescence Microscopy for Mitochondrial Mass Analysis

The cells were grown on coverslips previously coated with 2% gelatin with or without L-glutamine for 12 days and then stained with MitoTracker Red according to the manufacturer’s instructions (Molecular Probes Inc.). Fluorescence signals were analyzed by recording stained 63x and 100x images (shown here as 100x) using a CCD camera (Zeiss). The red areas were manually selected, and the percentage for each analyzed sample was calculated.

### mRNA analysis for mitochondrial codified ETC proteins

Total RNA was extracted from 15 VHM and 12 NHM cultures using the Aurum Total mini kit (Biorad). Next, cDNA was synthetized from 1 μg of total RNA using the FirstAid kit (Fermentas) and amplified in a reaction mixture containing iQSYBR Green Supermix (Biorad) and 25 pmol of forward and reverse primers using an iQ5 Light Cycler (Biorad). All samples were run in triplicate, and the relative expression was determined by normalizing the results against actin mRNA. Due to the importance of the internal control chosen for sample normalization, comparative analyses were randomly performed using the commonly used housekeeping gene GAPDH (data not shown).

### mtDNA quantification

Total DNA was prepared from melanocytes using DNeasy Blood and Tissue (Qiagen) according to the manufacturer’s recommendations and stored at −20 °C. mtDNA content was measured by real-time PCR using an iQ5 real-time PCR (BioRad). Amplification conditions were as follows: 5 min at 95 °C, then 45 cycles of 15 s at 95 °C and 1 min at 58 °C. A dissociation curve was also calculated for each sample to ensure presence of a single PCR product. The experiment was performed in triplicate. As previously reported (Moiseeva *et al*., 2009), the relative quantification of mitochondrial DNA (mtDNA) over nuclear DNA (nuDNA) levels was determined using the difference in the threshold cycle values of nuclear TATA-box-binding protein region on chromosome 6 and the mitochondrial non-coding control region D-loop (ΔCt, namely, Ct_mtDNA_ − Ct_nuDNA_). The relative abundance of the mitochondrial genome was reported as 2^−ΔCt^. The primers used were the following: mtDNA forward, GATTTGGGTACCACCCAAGTATTG; reverse, GTACAATATTCATGGTGGCTGGCA; and nuDNA forward, TTCCACCCAAGTATTG; reverse, TGTTCCATGCAGGGGAAAACAAGC.

### Statistical Analysis

We used Student’s T test for all the performed assays.
